# Activation of necroptosis in human and experimental cholestasis

**DOI:** 10.1038/cddis.2016.280

**Published:** 2016-09-29

**Authors:** Marta B Afonso, Pedro M Rodrigues, André L Simão, Dimitry Ofengeim, Tânia Carvalho, Joana D Amaral, Maria M Gaspar, Helena Cortez-Pinto, Rui E Castro, Junying Yuan, Cecília M P Rodrigues

**Affiliations:** 1 Research Institute for Medicines (iMed.ULisboa), Faculty of Pharmacy, Universidade de Lisboa, Lisbon, Portugal; 2 Department of Cell Biology, Harvard Medical School, Boston, MA, USA; 3 Histology and Comparative Pathology Laboratory, Instituto de Medicina Molecular, Lisbon, Portugal; 4 Department of Gastrenterology, Hospital Santa Maria, Lisbon, Portugal; 5 Faculty of Medicine, Universidade de Lisboa, Lisbon, Portugal

## Abstract

Cholestasis encompasses liver injury and inflammation. Necroptosis, a necrotic cell death pathway regulated by receptor-interacting protein (RIP) 3, may mediate cell death and inflammation in the liver. We aimed to investigate the role of necroptosis in mediating deleterious processes associated with cholestatic liver disease. Hallmarks of necroptosis were evaluated in liver biopsies of primary biliary cholangitis (PBC) patients and in wild-type and RIP3-deficient (RIP3^−/−^) mice subjected to common bile duct ligation (BDL). The functional link between RIP3, heme oxygenase-1 (HO-1) and antioxidant response was investigated *in vivo* after BDL and *in vitro*. We demonstrate increased RIP3 expression and mixed lineage kinase domain-like protein (MLKL) phosphorylation in liver samples of human PBC patients, coincident with thioflavin T labeling, suggesting activation of necroptosis. BDL resulted in evident hallmarks of necroptosis, concomitant with progressive bile duct hyperplasia, multifocal necrosis, fibrosis and inflammation. MLKL phosphorylation was increased and insoluble aggregates of RIP3, MLKL and RIP1 formed in BLD liver tissue samples. Furthermore, RIP3 deficiency blocked BDL-induced necroinflammation at 3 and 14 days post-BDL. Serum hepatic enzymes, fibrogenic liver gene expression and oxidative stress decreased in RIP3^−/−^ mice at 3 days after BDL. However, at 14 days, cholestasis aggravated and fibrosis was not halted. RIP3 deficiency further associated with increased hepatic expression of HO-1 and accumulation of iron in BDL mice. The functional link between HO-1 activity and bile acid toxicity was established in RIP3-deficient primary hepatocytes. Necroptosis is triggered in PBC patients and mediates hepatic necroinflammation in BDL-induced acute cholestasis. Targeting necroptosis may represent a therapeutic strategy for acute cholestasis, although complementary approaches may be required to control progression of chronic cholestatic liver disease.

Cholestasis is a pathological condition characterized by disruption of bile flow, resulting in intrahepatic and systemic retention of bile acids, with a concomitant toxic response in liver parenchymal cells, inflammation, progression to fibrosis and, ultimately, cirrhosis and premature death. Cholestatic liver injury may arise from a large number of inflicting insults, including genetic disorders, drug toxicity, hepatobiliary malignancies or obstruction of the biliary tract.^1^ Liver transplantation remains one of the few available options for these patients.^2^ This calls for novel therapeutic approaches, based in a better understanding of molecular, cellular and biochemical mechanisms underlying pathogenesis of cholestasis.

Inappropriate activation of cell death is intimately associated with the pathogenesis of cholestatic liver diseases.^3^ In addition to apoptosis, different regulated necrotic cell death routines are emerging, defined as genetically controlled cell death processes with morphological hallmarks of oncotic necrosis.^4^ Necroptosis, the most well-studied pathway of regulated necrosis, depends on receptor-interacting protein (RIP) 3 kinase activity. In particular conditions, RIP1 and RIP3 engage in physical interactions upon activation of death receptors,^5^ creating a filamentous amyloid protein complex called necrosome.^6^ Upon phosphorylation by active RIP3, mixed lineage kinase domain (MLKL) oligomerizes and translocates to cellular membranes, hence compromising their ability to preserve ionic homeostasis.^7, 8^

Activation of necroptosis appears to constitute a pathophysiological event in chronic inflammatory liver diseases, namely alcoholic and non-alcoholic steatohepatitis (NASH).^9, 10, 11^ Although controversial,^12^ necroptosis has also been suggested to mediate experimental acetaminophen-induced hepatotoxicity in early phases,^13, 14^ and phosphorylated MLKL (p-MLKL) is detected in liver biopsies of patients with drug-induced liver injury (DILI),^7^ frequently associated with cholestasis.^15^ In agreement with a role of necroptosis in cholestatic liver injury, combined ablation of hepatocyte-specific caspase-8 and nuclear factor-*κ*B essential modulator results in spontaneous massive liver necrosis and cholestasis in mice, with a concomitant formation of necrosome complexes in the foci of necrotic areas.^16^ Further, in an animal model of chronic hepatitis and severe cholestasis, absence of RIP3 attenuates cholestasis and jaundice, suggesting the involvement of RIP3 signaling in cholestasis.^17^

In this study, we provide evidence of hallmarks of necroptosis activation in human primary biliary cholangitis (PBC) liver tissue. Further, we show that, in mice subjected to common bile duct ligation (BDL), genetic ablation of RIP3 protects hepatocytes from oxidative stress, inflammation and necrosis, but fails to prevent BDL-induced secondary fibrosis.

## Results

### Necroptosis is activated in the liver of patients with PBC

**Figure 1:**
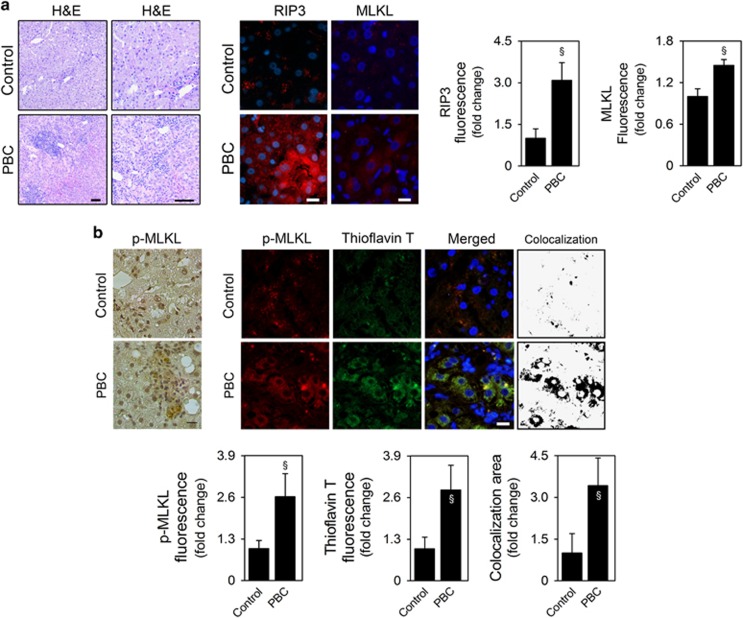
Necroptosis is activated in the liver of patients with PBC. (**a**) Representative H&E-stained sections of liver from control subjects (*n*=5) and PBC patients (*n*=5); scale bar, 100 *μ*m (left). Representative RIP3 and MLKL immunofluorescence (red) in liver from control and PBC patients; scale bar, 10 *μ*m (right). (**b**) Representative immunoperoxidase staining for p-MLKL in human liver samples. Scale bar, 50 *μ*m (left). Representative p-MLKL immunofluorescence (red) and thioflavin T staining (green) in liver from control and PBC patients. Scale bar, 10 *μ*m (right). Nuclei were counterstained with Hoechst 33258 (blue). Histograms show the quantification of RIP3, total MLKL, p-MLKL and thioflavin T mean fluorescence intensity and quantification of colocalization area of thioflavin T and p-MLKL, as described in Materials and Methods section. Quantification was performed in at least eight high-power fields per liver sample. Data are expressed as mean±S.E.M. fold change. ^§^*P*<0.05 from Control

PBC is characterized by gradual destruction of small intrahepatic bile ducts, associated with portal inflammatory infiltration and cholangitis (Figure 1a). High levels of RIP3 and MLKL expression were detected in the liver of PBC patients, in contrast with its low hepatic expression in healthy controls (*P*<0.05; Figure 1a). In agreement with the amyloid structure of the necrosome, increased levels of RIP3 protein exhibited with a punctuated pattern in the cytoplasm of hepatic cells from PBC patients (Figure 1a and Supplementary Figure S1A). In addition, as previously reported in human NASH liver biopsies,^10^ RIP3 was often expressed in hepatocytes surrounded by lymphocytic infiltrates (Figure 1a), and in cells morphologically resembling biliary epithelia (Supplementary Figure S1A, lower panel), suggesting that necroptosis may also be involved in the destruction of small intrahepatic bile ducts, characteristic of this disease. Still, RIP3-dependent signaling may also mediate apoptosis^18, 19^ and has been shown to directly regulate inflammatory signaling.^20^ As such, we also evaluated p-MLKL expression in human liver samples by immunohistochemistry and performed double immunofluorescence staining of p-MLKL, a non-redundant executer of necroptosis, and thioflavin T, a marker of *β*-amyloid fibrils. Similarly to RIP3, p-MLKL was increased in liver parenchymal cells of PBC patients, compared with healthy controls, where p-MLKL expression was absent (*P*<0.05; Figure 1b and Supplementary Figure S1B). Of note, p-MLKL and MLKL fluorescence increased by 2.6- and 1.5-fold in PBC patients, respectively, compared with healthy controls, further suggesting MLKL phosphorylation. Finally, p-MLKL colocalized with thioflavin T staining (*P*<0.05; Figure 1b), indicating that MLKL was phosphorylated in the presence of amyloid aggregates, characteristic of necrosome complexes.^6^ Altogether, our results indicate that necroptosis is activated in liver parenchymal cells of PBC patients, likely contributing to cholestasis and liver injury.

### Necroptosis is activated in the liver of BDL mice

**Figure 2:**
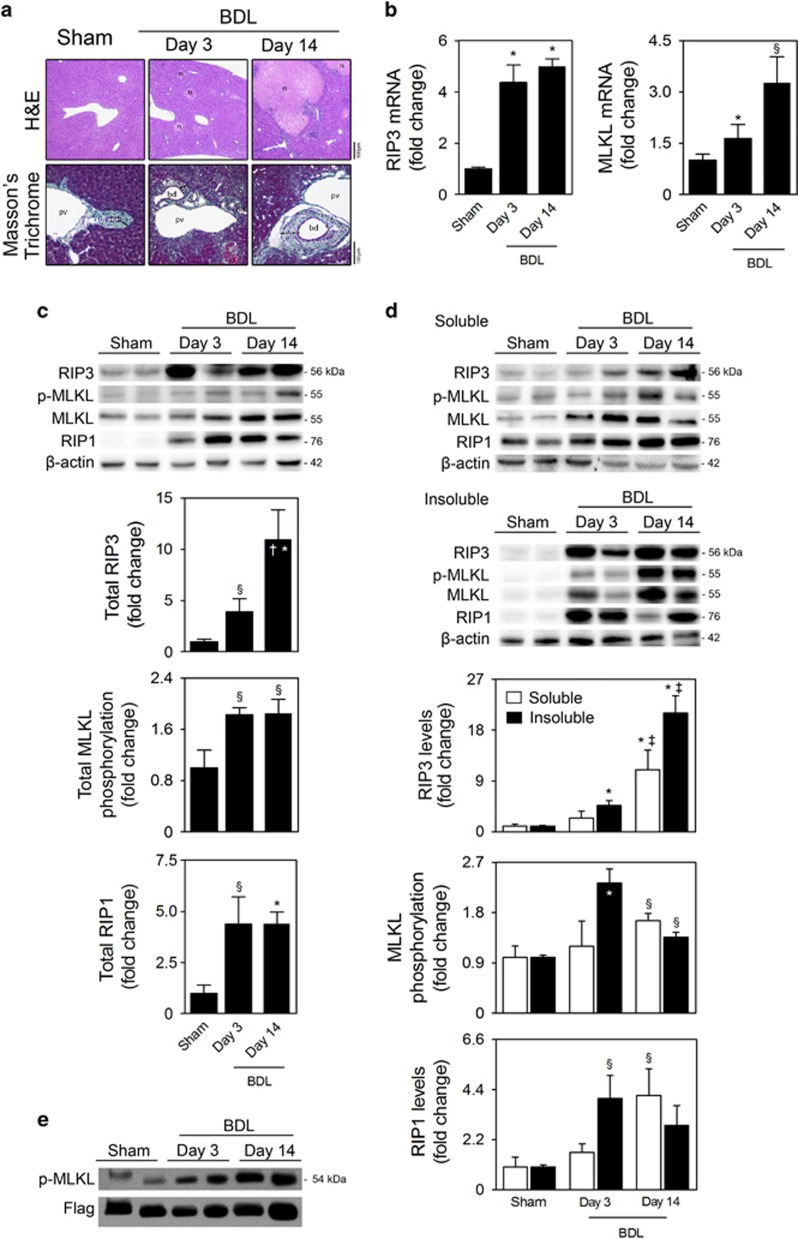
Necroptosis is activated in the liver of mice after BDL. C57BL/6N mice were subjected to sham or BDL surgical procedures and killed at days 3 and 14. (**a**) Representative H&E and Massons' Trichrome-stained liver sections. (**b**) qRT-PCR analysis of RIP3 and MLKL in mouse liver. (**c**) Immunoblotting and densitometry of total RIP3, p-MLKL, MLKL and RIP1. (**d**) RIP3, MLKL and RIP1 in insoluble and soluble fractions of liver whole-cell lysates. Blots of RIP3, MLKL and RIP1 were normalized to endogenous *β*-actin, whereas p-MLKL was normalized to MLKL. Representative immunoblots are shown. Results are expressed as mean±s.e.m. fold change of 7–10 individual mice. ^§^*P*<0.05 and *P<0.01 from respective sham-operated mice; ^†^*P*<0.05 and ^‡^*P*<0.01 from respective BDL day 3. (**e**) Representative *in vitro* RIP3 kinase activity from sham and BDL mice

BDL in mice was associated with multifocal hepatocellular necrosis and inflammatory cell infiltration, mainly seen at the necrotic areas. At later stages (day 14 post-BDL), bile duct hyperplasia and periportal fibrosis were also observed (Figure 2a). Concomitantly, we found that mRNA and protein expression of RIP3 and MLKL, as well as MLKL phosphorylation, strongly increased in the liver of BDL mice, comparing with sham mice, (at least, *P*<0.05; Figures 2b and c). Although RIP1 mRNA levels remained unchanged (data not shown), RIP1 protein levels were also increased in whole-liver cell lysates from BDL mice (at least, *P*<0.05; Figure 2c).

We previously reported that the evaluation of RIP3 and MLKL in soluble/insoluble protein fractions constitutes a suitable tool for identification of ongoing necroptosis in an experimental disease condition.^11, 21^ In fact, although RIP3 and MLKL progressively increased with time in both liver soluble and insoluble protein fractions of BDL mice, they were preferentially retained in the insoluble fraction (Figure 2d). The p-MLKL/MLKL ratio is particularly increased in insoluble fractions at day 3 post-BDL, indicating that necroptosis is an early event in the BDL murine model. Curiously, RIP1 was particularly sequestered in insoluble fractions at early time-points (*P*<0.05), in agreement with the concept that RIP1 is likely the upstream kinase that initiates necrosis signaling, after which RIP3 autophosphorylation and subsequent recruitment of MLKL propagate necroptosis.^22^ We also immunoprecipitated RIP3 from liver extracts of sham and BDL mice and performed an *in vitro* RIP3 kinase assay using purified human MLKL as a substrate. Accordingly, this assay demonstrated that phosphorylation of MLKL increased in the liver of BDL mice (Figure 2e). Overall, our results strongly indicate that necroptosis is activated in the liver of mice upon BDL, and positively correlate with BDL-induced hepatic damage, suggesting a role of necroptosis in the pathogenesis of cholestatic liver injury.

### RIP3 deficiency prevents necroinflammation induced by BDL in mice

**Figure 3:**
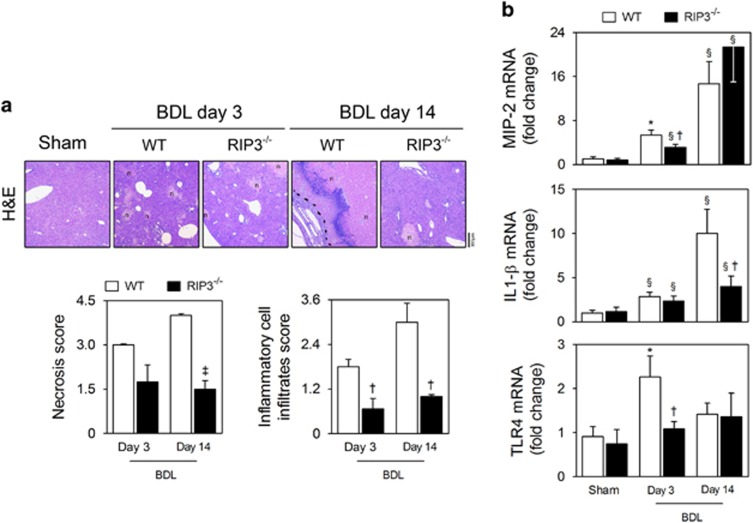
RIP3 deficiency ameliorates hepatic necroinflammation in the BDL murine model. C57BL/6N WT and RIP3^−/−^ mice were subjected to sham or BDL surgical procedures and killed at days 3 and 14. (**a**) Representative images of H&E-stained liver sections (top). Necrosis and inflammatory cell infiltration was scored as described in Materials and Methods section (bottom). Results are expressed as mean±s.e.m. fold change of 4–5 individual mice. (**b**) qRT-PCR analysis of *MIP-2*, *IL-1β* and *TLR4* in mouse liver. Results are expressed as mean±S.E.M. fold change of 7–10 individual mice. ^§^*P*<0.05 and *P<0.01 from sham-operated mice; ^†^*P*<0.05 and ^‡^*P*<0.01 from BDL WT mice at respective time-point

C57BL/6N wild-type (WT) or RIP3-deficient (RIP3^−/−^) mice were subjected to sham surgery or BDL, and assessed for 3 and 14 days. At day 3, hepatocellular necrosis in RIP3^−/−^ mice tended to be less severe than in WT mice, although without reaching statistical significance (Figure 3a); however, at day 14, absence of RIP3 was significantly associated with decreased hepatocellular damage (*P*<0.01; Figure 3a). These results suggest that necroptotic cell death may contribute to the extent of bile infarcts during progression of BDL-induced liver injury. RIP3 deficiency was also associated with decreased inflammatory cell infiltration induced by BDL at both early and late time-points (*P*<0.05; Figure 3a). Further, absence of RIP3 appears to delay the progressive increase in hepatic macrophage inflammatory protein 2 (MIP-2) mRNA expression induced by BDL and reduced mRNA levels of other pro-inflammatory mediators, namely interleukin (IL) 1-*β* and Toll-like receptor 4 (TLR4), compared with BDL WT mice (*P*<0.05; Figure 3b). These findings demonstrate that deletion of RIP3 ameliorates necroinflammation associated with experimental obstructive cholestasis, particularly at early stages.

### RIP3 deficiency does not affect BDL-induced fibrosis and apoptosis

**Figure 4:**
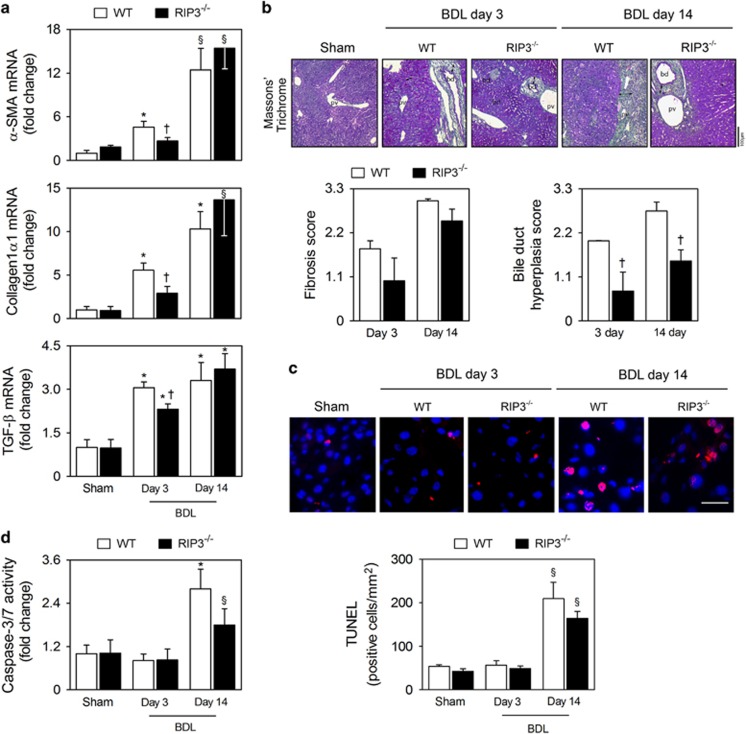
Deletion of RIP3 does not improve BDL-induced fibrosis and apoptosis. C57BL/6N WT and RIP3^−/−^ mice were subjected to sham or BDL surgical procedures and killed at days 3 and 14. (**a**) qRT-PCR analysis of *α-SMA*, *collagen-1α1* and *TGFβ* in mouse liver. Results are expressed as mean±S.E.M. fold change of 7–10 individual mice. (**b**) Representative images of Masson's Trichrome-stained liver sections (top). Periportal fibrosis and bile duct hyperplasia were scored as described in Materials and Methods section (bottom). Results are expressed as mean±s.e.m. fold change of 4–5 individual mice. (**c**) TUNEL staining of liver tissue sections. Nuclei were counterstained with Hoechst 33258 (blue). Scale bar, 30 *μ*m (left). Histograms show the quantification of TUNEL-positive cells/mm^2^ (right). Results are expressed as mean±S.E.M. fold change of 4–5 individual mice. (**d**) Caspase-3/7 activity assay. ^§^*P*<0.05 and *P<0.01 from sham-operated mice; ^†^*P*<0.05 from BDL WT mice at respective time-point

*α*-Smooth muscle actin (*α*-SMA) mRNA levels, a marker for hepatic stellate cell activation, and collagen-1*α*1 were progressively increased with time in BDL murine model (at least, *P*<0.05; Figure 4a). RIP3 deficiency decreased mRNA levels of *α*-SMA and collagen-1*α*1 by almost 50% at day 3 (*P*<0.05), but did not significantly affect mRNA expression at 14 days after BDL. Similar results were obtained when analyzing transforming growth factor (TGF)-*β* mRNA levels, a profibrogenic mediator (Figure 4a). We further tested for the presence of periportal fibrosis through Masson's Trichrome staining in liver sections from WT and RIP3^−/−^ mice, post-BDL. This showed a mild reduction in periportal fibrosis in RIP3^−/−^, compared with WT mice. However, at later stages, no clear difference was seen between RIP3 and WT mice, indicating that RIP3 deficiency fails to prevent fibrosis (Figure 4b), corroborating the data obtained with the markers of hepatic fibrosis. Curiously, it has been demonstrated that cholangiocyte proliferation contributes to initiation and progression of peribiliary fibrosis,^3^ and our results show that absence of RIP3 associates with significantly decreased bile duct hyperplasia at both 3 and 14 days post-BDL (*P*<0.05; Figure 4b).

Apoptosis may promote fibrogenesis upon cholestasis,^23^ whereas inhibition of one cell death pathway may activate alternative cell death routines.^11, 17^ Thus, we sought to elucidate whether compensatory liver apoptosis was evident in RIP3^−/−^ mice following BDL. We found that the number of TUNEL-positive cells in the liver increased ~4-fold at day 14 post-BDL in WT mice (*P*<0.05), but was similar to sham-operated mice at day 3 (Figure 4c). In parallel, caspase-3/-7 activity increased ~2.5-fold at 14 days after BDL (*P*<0.01) and remained at basal levels at day 3 (Figure 4d). These data indicate that apoptosis is not triggered at early time-points in this BDL murine model, but is activated 14 days after BDL in WT mice, reflecting the peak of liver fibrosis. Of note, BDL-induced apoptosis at day 14 was not significantly affected by RIP3 deficiency (Figures 4c and d). Collectively, our results indicate that inhibition of necroptosis fails to prevent BDL-induced secondary liver fibrosis and apoptosis.

### Deletion of RIP3 decreases BDL-induced acute liver injury but aggravates cholestasis during experimental chronic cholestasis

WT mice displayed markedly increased serum alanine aminotransferase (ALT), aspartate aminotransferase (AST), alkaline phosphatase (AP), and total and conjugated bilirubin levels, at early and late time-points post-BDL, compared with sham mice (at least, *P*<0.05; Table 1). No difference was seen between sham WT and RIP3^−/−^ mice, for any of the above biochemical parameters. Strikingly, upon BDL, serum levels of ALT and AST were significantly reduced in RIP3^−/−^ mice compared with WT mice (*P<*0.05), at day 3 post-BDL, without any rescue for the hyperbilirubinemia and ALP elevation, indicating that the benefit of RIP3 deficiency in acute liver injury cannot be ascribed to changes in cholestasis. On the contrary, 14 days post-BDL, absence of RIP3 had no significant impact on AST and ALT levels, but was associated with increased AP and conjugated bilirubin levels, compared with WT mice (*P*<0.05; Table 1). Taken together, these results suggest that RIP3 deficiency decreases acute hepatocellular injury in the BDL murine model, but exacerbates cholestasis in animals with chronic obstructive cholestasis.

### RIP3 deficiency associates with increased hepatic HO-1 expression and iron accumulation in BDL mice

**Figure 5:**
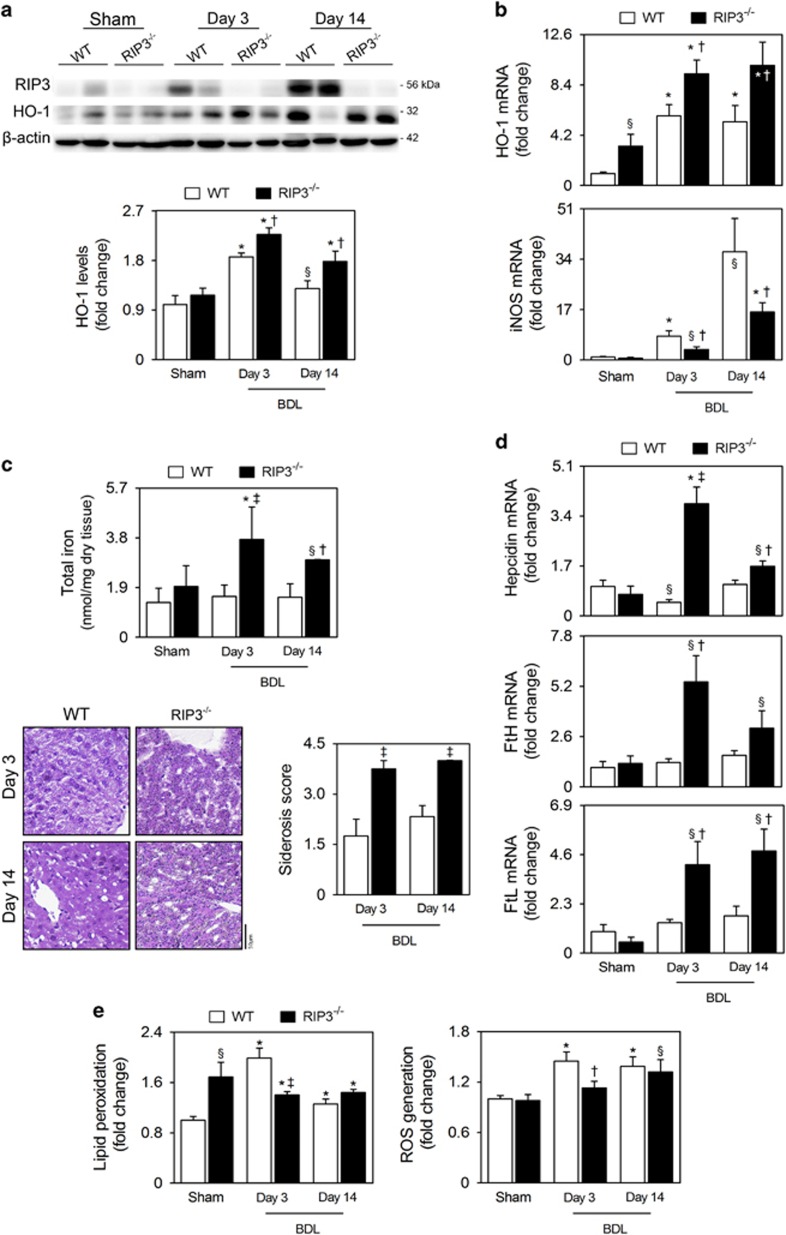
RIP3^−/−^ BDL mice display differential hepatic expression of HO-1, iron accumulation and oxidative stress. C57BL/6N WT and RIP3^−/−^ mice were subjected to sham or BDL surgical procedures and killed at days 3 and 14. (**a**) Immunoblotting and densitometry RIP3 and HO-1. Blots were normalized to endogenous *β*-actin. Representative immunoblots are shown. (**b**) qRT-PCR analysis of *HO-1* and *iNOS* in mouse liver. (**c**) Labile iron content in whole-liver lysates measured as described in Materials and Methods section. Results are expressed as mean±s.e.m. fold change of 7–10 individual mice (top). Representative images of Perls' Prussian Blue-stained liver sections. Iron accumulation score was performed as described in Materials and Methods section (bottom). Results are expressed as mean±s.e.m. fold change of 4–5 individual mice. (**d**) qRT-PCR analysis of *hepcidin, FtH* and *FtL* in mouse liver. (**e**) Malondialdehyde content in whole-liver lysates as a surrogate of lipid peroxidation measured as described in Materials and Methods section (left). Fluorescence intensity from whole-cell lysates stained with the fluorescent probe H_2_DCFDA was measured (right). Values are corrected with total protein content. Results are expressed as mean±S.E.M. fold change of 7–10 individual mice. ^§^*P*<0.05 and *P<0.01 from sham-operated mice; ^†^*P*<0.05 and ^‡^*P*<0.01 from BDL WT mice at respective time-point

Heme oxygenase-1 (HO-1) is an inducible enzyme that catalyzes the oxidative degradation of heme into equimolar amounts of biliverdin, labile iron and carbon monoxide. Biliverdin is then rapidly converted to bilirubin by the action of biliverdin reductase. As such, bilirubin, which we found increased in serum of RIP3^−/−^ mice 14 days after BDL, is one of the main products of HO-1. As previously reported for rats,^24, 25, 26^ mRNA and protein expression levels of HO-1 increased in livers of BDL WT mice, at both 3 and 14 days, compared with sham-operated mice (*P*<0.01). Importantly, hepatic expression of HO-1 was further and significantly increased in RIP3^−/−^ mice, compared with WT mice (*P*<0.05; Figures 5a and b). In addition, although BDL progressively increased inducible nitric oxide synthase (iNOS) mRNA expression in both genotypes, compared with sham controls (at least, *P*<0.05), BDL RIP3^−/−^ mice displayed reduced mRNA levels of iNOS compared with BDL WT mice (*P*<0.05; Figure 5b), consistent with the notion that HO-1 attenuates the expression of iNOS.^26, 27, 28^ Quantification of labile iron and the *Perls' Prussian Blue* staining also showed that deletion of RIP3 significantly increased iron deposition in livers from mice post-BDL at both time-points (at least, *P*<0.05; Figure 5c). Reflecting iron accumulation, BDL RIP3^−/−^ mice displayed increased hepatic expression of hepcidin, ferritin heavy chain (FtH) and ferritin light chain (FtL) (at least, *P*<0.05; Figure 5d).

Significantly higher levels of lipid peroxidation were detected in BDL WT and RIP3^−/−^ mice compared with WT sham mice at both time-points (*P*<0.01). Of note, lipid peroxidation at day 3 post-BDL in WT mice was higher than at day 14, suggesting that the antioxidant capacity of hepatocytes may partially decrease levels of lipid peroxides from day 3 onward (Figure 5e). Supporting this finding, it was reported that oxidative stress is more relevant during the early stage of biliary atresia.^29^ In turn, RIP3 deficiency significantly reduced lipid peroxidation at day 3 post-BDL (*P*<0.01), but not at day 14, compared with BDL WT mice. Curiously, lipid peroxidation was significantly increased in sham-operated RIP3^−/−^ mice compared with sham WT controls (*P*<0.05; Figure 5d). Finally, BDL induced reactive oxygen species (ROS) generation in WT mice at both time-points, compared with sham-operated mice (*P*<0.01), whereas deletion of RIP3 abrogated ROS production only at day 3 post-BDL (Figure 5e).

Overall, we show that deletion of RIP3 in BDL-induced cholestasis associates with increased hepatic HO-1 expression and liver siderosis. Further, RIP3 deficiency protects mice livers from oxidative stress only during the acute phase of BDL-induced cholestasis.

### HO-1 activation sensitizes RIP3-deficient hepatocytes to bile acid cytotoxicity

**Figure 6:**
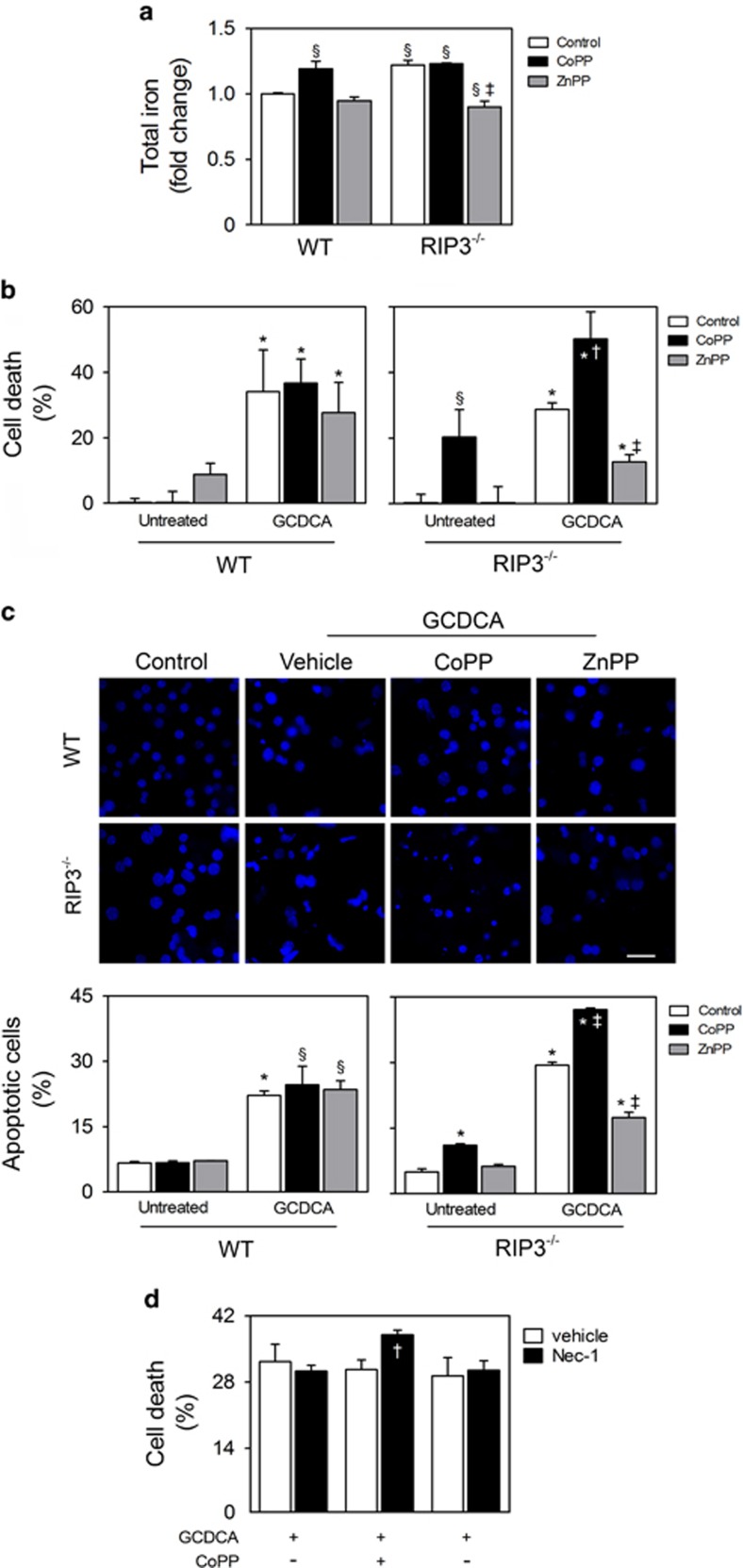
HO-1 is involved in iron accumulation and cytotoxic effects of GCDCA in RIP3-deficient primary mouse hepatocytes. Primary mouse hepatocytes isolated from WT and RIP3^−/−^ C57BL/6N mice were treated with CoPP (10 *μ*M), ZnPP (15 *μ*M) or vehicle control. After 1 h, cells were exposed to either GCDCA (50 *μ*M) or vehicle control. After 24 h, cells were harvested for total iron or immunoblotting. Cell death assays were performed after 48 h. (**a**) Total iron levels in whole cells were measured as described in Materials and Methods section. Values were normalized with total protein concentration. (**b**) Percentage of general cell death in primary mouse hepatocytes as assessed by LDH activity assay. (**c**) Apoptotic cells detected by Hoechst staining. Results are expressed as percentage of apoptotic cells (bottom). Representative images of untreated WT and RIP3^−/−^ primary hepatocytes and incubated with GCDCA, GCDCA plus CoPP or GCDCA plus ZnPP are shown. Scale bar, 30 *μ*M. (**d**) Primary mouse hepatocytes isolated from WT and RIP3^−/−^ C57BL/6N mice were treated with Nec-1 (100 *μ*M) or vehicle control. After 1 h, cells were exposed to GCDCA (50 *μ*M) for 48 h. Percentage of general cell death was as assessed by the LDH activity assay. Results are expressed as mean±S.E.M. fold change or percentage from three independent cultures from each genotype. ^§^*P*<0.05 and *P<0.01 from Control; ^†^*P*<0.05 and ^‡^*P*<0.01 from respective control

The fact that RIP3 deficiency associates with induction of HO-1 expression and iron accumulation in the liver of BDL mice prompted us to further determine the mechanistic link between iron accumulation and HO-1 activity in primary hepatocytes isolated from WT and RIP3^−/−^ mice. In fact, cellular levels of total iron were significantly increased in RIP3^−/−^ primary mouse hepatocytes, as compared with WT hepatocytes (*P*<0.05; Figure 6a). Furthermore, levels of iron were significantly elevated in WT primary mouse hepatocytes loaded with the HO-1 inducer cobalt protoporphyrin (CoPP) (*P*<0.05), whereas zinc protoporphyrin-9 (ZnPP), a HO-1 chemical inhibitor, reduced the levels of iron in RIP3^−/−^ hepatocytes (*P*<0.01; Figure 6a). Thus, the constitutive induction of HO-1 in RIP3^−/−^ mouse hepatocytes promotes iron accumulation.

The hydrophobic bile acid glycochenodeoxycholic acid (GCDCA) is a major constituent of human serum and bile during cholestasis. In order to evaluate whether HO-1 activation could directly modulate hepatocyte cell death pathways in the context of bile acid toxicity, primary hepatocytes isolated from WT and RIP3^−/−^ mice were incubated with GCDCA in the presence or absence of CoPP or ZnPP. GCDCA increased overall cell death and apoptosis in both WT and RIP3^−/−^ primary mouse hepatocytes (*P*<0.01). Pre-treatment of WT primary hepatocytes with CoPP or ZnPP did not impact on GCDCA-induced cytotoxicity. Nevertheless, although ZnPP protected RIP3^−/−^ primary hepatocytes from GCDCA-induced cell death (*P*<0.01), CoPP was cytotoxic *per se* and enhanced the sensitivity to overall cell death induced by GCDCA (*P*<0.05; Figures 6b and c). The interplay between RIP3 deficiency and HO-1 is reinforced by the decreased expression of HO-1 induced by GCDCA in WT mouse hepatocytes (*P*<0.05) but not in RIP3^−/−^ mouse hepatocytes (Supplementary Figure S2). We further evaluated whether inhibition of RIP1 kinase activity using necrostatin-1 (Nec-1) affected GCDCA-induced cytotoxicity. Although Nec-1 slightly increased the toxicity of GCDCA plus CoPP (*P*<0.05), co-incubation of Nec-1 and ZnPP failed to protect WT mouse hepatocytes from GCDCA-induced cell death. Further, Nec-1 alone had no effect on GCDCA toxicity in WT mouse hepatocytes, indicating that in these experimental conditions necroptosis is not triggered by GCDCA (Figure 6d). In agreement, RIP3 and p-MLKL were not detected in primary mouse hepatocytes treated with GCDCA for 24 h (data not shown). Altogether, these results indicate that, in contrast to WT cells, cytotoxicity in RIP3^−/−^ primary hepatocytes associates with induction of HO-1.

## Discussion

Upregulation of RIP3 is a common pathological feature in a range of human inflammation-driven liver diseases, namely hepatitis B and C, alcoholic liver disease, NASH, DILI and autoimmune hepatitis,^9, 10, 11, 14^ likely promoting inflammation in a necroptosis-dependent and -independent manner.^30^ Our results are the first to show that RIP3 and p-MLKL are simultaneously activated in liver parenchymal cells of PBC patients, suggesting that necroptosis likely has an active role in disease triggering and progression. Further, in an animal model of obstructive cholestasis, the necrotic cell death characteristic of BDL-induced bile infarcts is, at least in part, necroptosis. However, whether necroptosis is induced by bile acids or other mechanisms, such as inflammation, is still not clear. For instance, our results show that murine TLR4 is upregulated early in BDL. Activation of TLR4 in liver parenchymal cells may trigger necroptosis by activating RIP3 through Toll or IL-1 receptor (TIR) domain-containing adaptor inducing interferon-*β* (TRIF).^31^

HO-1 is a well-established stress-inducible enzyme,^32^ found upregulated in the liver of BDL rats.^25, 33, 34^ Overexpression of HO-1 can protect steatotic livers from ischemia–reperfusion injury in rats^35^ and from chronic ethanol-induced liver injury in mice.^36^ Interestingly, HO-1 upregulation reduces hepatic RIP3 expression in mice upon chronic ethanol feeding and protects primary rat hepatocytes from necroptosis induced by co-exposure of ethanol and tumor necrosis factor-*α* (TNF-*α*).^36^ Conversely, here, we showed that RIP3 deficiency is associated with increased HO-1 expression. These findings suggest a mechanistic loop involving HO-1 and RIP3 that is warranted to be further explored in the future. Nevertheless, detrimental effects associated with chronic HO-1 upregulation could also be present in RIP3^−/−^ mice following BDL;^25, 33, 34^ RIP3 deficiency exacerbated cholestasis and jaundice 14 days after BDL, as evidenced by increased levels of bilirubin and AP, likely due to sustained overexpression of HO-1. Indeed, HO-1 is a rate-limiting enzyme in the bilirubin metabolism and it directly worsens hyperbilirubinemia in BDL rats.^37^

BDL is a surgical model for severe obstructive cholestasis that results in marked jaundice and hepatocellular damage.^38^ Inhibition of necroptosis in other chronic cholestatic contexts may carry therapeutic benefits in preventing cholestasis. Indeed, RIP3 deficiency attenuated development of jaundice and cholestasis in severe chronic hepatitis induced by conditional deletion of TGF-*β*-activated kinase-1 in liver parenchymal cells, although aggravating hepatitis.^17^ Finally, HO-1 has been suggested to stimulate bile flow during ethinylestradiol-induced cholestasis in rats.^39^

Liver fibrosis is a critical endpoint of murine models reflecting chronic cholestatic conditions. Here, we found that inhibition of necroptosis failed to prevent liver fibrosis. Accordingly, it has been demonstrated that, in contrast to apoptosis, necroptosis was not implicated in fibrogenesis in an animal model of severe hepatitis and cholestasis.^17^ Indeed, our results show that apoptosis was activated late after BDL in parallel with development of liver fibrosis, whereas it is widely accepted that apoptosis may trigger hepatic fibrogenesis.^23^ For instance, hepatic stellate cell activation and fibrosis are reduced in TNF-related apoptosis-inducing ligand knockout mice following BDL for 14 days, likely secondary to diminished apoptosis.^40^ In addition, pharmacologic inhibition of caspases also protected against cholestatic hepatocyte injury and decreased stellate cell activation and fibrosis 10 days after BDL in mice.^41^ Therefore, in the context of the BDL murine model, apoptosis may be a stronger trigger of fibrogenesis compared with necroptosis.

In theory, a remarkable reduction in chronic liver injury should translate into reduced hepatic fibrosis, in part because chronic inflammation represents a fundamental factor connecting liver injury with hepatic fibrosis. Here, we demonstrated that, although RIP3 deficiency decreases hepatic expression of fibrogenic markers at 3 days after BDL, and strongly diminishes histological evidence of hepatocellular damage and inflammation during the entire BDL time-course, it is not sufficient to ameliorate liver fibrosis in mice with chronic obstructive cholestasis. This may be attributed to sustained upregulation of hepatic HO-1 expression in RIP3^−/−^ mice. Indeed, HO-1 overexpression significantly exacerbates liver fibrosis and portal hypertension induced by chronic BDL in rats.^25, 33, 34^ Further, and in agreement with our results, chronic HO-1 overexpression directly promotes hepatic iron accumulation in BDL rats,^25, 37^ whereas iron chelation diminishes liver fibrosis.^37^ We found that RIP3^−/−^ primary hepatocytes displayed increased iron levels compared with WT hepatocytes, being iron content modulated by HO-1 activity. In addition, we confirmed the toxic effect of HO-1 induction and its involvement in the sensitization of RIP3^−/−^ primary mouse hepatocytes to bile acid-induced cytotoxicity. In fact, it is well established that chronic iron overload aggravates hepatic stellate cell activation, whereas progression to fibrosis positively correlates with exposure to iron in hereditary hemochromatosis.^42, 43^ Of note, our results showed that, in parallel with HO-1 overexpression, BDL RIP3^−/−^ mice displayed improved parameters of oxidative stress and fibrosis at early time-points, together with increased hepatic siderosis. Noteworthy, HO-1 induction can ameliorate immune liver fibrosis in rats^44^ and in multidrug resistance transporter 2 knockout mice, a murine model of chronic cholestasis, inflammation and biliary fibrosis.^45^ In light of the above, induction of HO-1 in RIP3^−/−^ mice may be hepatoprotective and anti-fibrogenic under particular conditions, but sustained activation could have detrimental effects. Thus, further pre-clinical research is needed to fully elucidate the multiple facets of necroptosis inhibition and its relation with HO-1 activation in arresting progression of chronic liver disease.

In conclusion, RIP3-dependent signaling is triggered in human PBC and mediates hepatic necroinflammation in BDL-induced liver damage in mice. Moreover, inhibition of necroptosis attenuates acute liver injury and oxidative stress in mice following BDL, but fails to prevent BDL-induced secondary hepatic fibrosis and is associated with enhanced chronic jaundice and cholestasis. Upregulation of HO-1 may explain the side effects associated with inhibition of RIP3-dependent signaling. Targeting necroptosis may provide an unprecedented opportunity to develop novel therapeutic strategies to attenuate acute cholestatic liver injury.

## Materials and Methods

### Patients

Liver tissue was prospectively and sequentially collected from patients who fulfilled the clinical and pathological diagnostic features of PBC. Liver biopsies from age and sex-matched controls were obtained from potential liver donors, with normal liver histology and biochemistry. All liver specimens were fixed and processed for routine diagnosis and were blindly evaluated by an experienced pathologist. The study and sample collection were performed after informed consent and Institutional Review Board approval by the Hospital de Santa Maria, Centro Hospitalar Lisboa Norte EPE, in accordance with the Declaration of Helsinki.

### Animal experiments

Common BDL^38^ was performed in 8–10 weeks C57BL/6N WT mice with 25–30 g (WT; Charles River Laboratories International, Inc., Wilmington, MA, USA) or in RIP3^−/−^ mice.^46^ Seven to 10 animals were included in each experimental group. Mice were anesthetized with isoflurane and laid on a heating pad. After disinfection of the skin, a midline abdominal skin and muscle incision, about 3 cm long, was performed to expose the xyphoid process. The cut (laparotomy) wall was retracted bilaterally to expose the liver. Using a moistened gauze tip, the middle and left lateral lobe were lifted to expose the hepatic hilum. A 5-0 non-absorbable synthetic monofilament was placed around the common bile duct using microdissection forceps, followed by double ligation. Middle and left lateral lobes were then gently placed into their anatomical location and the aponeurotic layer of the abdominal wall muscle closed with synthetic monofilament non-absorbable sutures. Skin abdominal incision was closed with wound clips. After closing the abdomen, the animal stayed on a warming pad for recovery during 15 min. The analgesic buprenorphine was administered by subcutaneous injection before the surgery and for 48 h after surgery to treat post-operative pain (0.05 mg/kg bw). Three or 14 days after surgeries, animals were killed by isoflurane overdose. Serum was collected and the liver removed; one lobe was collected, rinsed in normal saline and immediately flash frozen in liquid nitrogen for protein and RNA extraction; another lobe was included in optimal cutting temperature compound (4583, Tissu-Tek, Sakura, Alphen aan den Rijn, The Netherlands), and the final lobe fixed in paraformaldehyde (4%, wt/vol) in phosphate-buffered saline (PBS; Thermo Fisher Scientific, Inc., Waltham, MA, USA) for paraffin-embedded sectioning. Controls underwent sham operation with exposure but without ligation of the common bile duct. All animal experiments were carried out with the permission of the local animal ethical committee in accordance with the EU Directive (2010/63/EU), Portuguese law (DL 113/2013) and all relevant legislations. The experimental protocol was approved by Direcção Geral de Alimentação e Veterinária. Animals received humane care in a temperature-controlled environment with a 12- h light–dark cycle, complying with the Institute's guidelines, and as outlined in the 'Guide for the Care and Use of Laboratory Animals' prepared by the National Academy of Sciences and published by the National Institutes of Health (NIH publication 86-23 revised 1985).

### Histopathology and serum analyses

Paraffin-embedded sections (3-4 *μ*m) were stained with hematoxylin and eosin (H&E), Masson's Trichrome or Perls' Prussian Blue using standard protocols. Liver sections were scored in a blinded manner by an experienced pathologist, using a four-point severity scale (0, normal; 1, mild; 2, moderate; 3, severe), for necrosis, inflammatory cell infiltration, bile duct hyperplasia, fibrosis and iron deposition. Serum levels of ALT, AST, AP and total and conjugated bilirubin were determined using standard clinical chemistry techniques.

### Total and soluble/insoluble protein extraction and immunoblotting

Total and soluble/insoluble protein extraction was performed as previously described.^11^ Steady-state levels of RIP3, MLKL, RIP1 and HO-1 were determined by immunoblot analysis. Briefly, 50 *μ*g of total protein extracts, insoluble or soluble protein fractions was separated on an 8 or 10% sodium dodecyl sulfate-polyacrylamide gel electrophoresis (SDS-PAGE). Following electrophoretic transfer onto nitrocellulose membranes and blocking with 5% milk solution, blots were incubated overnight at 4 °C with primary rabbit polyclonal antibodies against RIP3 (1 : 1000, AHP1797, AbD Serotec, Bio-Rad Laboratories, Hercules, CA, USA), p-MLKL (1 : 1000, ab196436, Abcam plc, Cambridge, UK), MLKL (1 : 500, SAB1302339, Sigma-Aldrich Co., St. Louis, MO, USA), RIP1 (1 : 1000, D94C12, Cell Signaling, Inc., Danvers, MA, USA) and HO-1 (ADI-SPA-895, 1 : 1000, Enzo Life Sciences Inc., Farmingdale, NY, USA) and with a secondary antibody conjugated with horseradish peroxidase (Bio-Rad Laboratories) diluted 1 : 5000 in blocking solution for 1 h at room temperature. Membranes were processed for protein detection using Super Signal substrate (Pierce, Thermo Fisher Scientific Inc.). *β*-Actin (1 : 20 000; A5541; Sigma-Aldrich Co.) was used as loading control.

### *In vitro* RIP3 kinase activity

Two mg of frozen liver tissue was homogenized in 1 ml of ice-cold Nonidep P-40 (NP-40) lysis buffer (25 mM hepes pH 7.5, 120 mM NaCl, 0.27 M sucrose, 5 mM EDTA, 5 mM EGTA, 0.2% NP-40, 50 mM NaF, 10 mM *β*-glycerophosphatase, 5 mM sodium pyrophosphate), supplemented with 1 mM Na_3_VO_4_, 1 mM benzamide, 0.1% *β*-mercaptoethanol, 0.1 M phenylmethanesulfonyl fluoride and 1 x complete protease inhibitor tablet diluted 1 : 100 (Roche Diagnostics GmbH, Mannheim, Germany). Immunoprecipitation of RIP3 was carried out using 2 *μ*g of anti-RIP3 antibody (AbD Serotec) and 20 *μ*l of protein A/G-sepharose beads for 4 h at 4 °C. Following three washes with NP-40 lysis buffer and two washes with buffer A (25 mM Hepes pH 7.5, 0.1 *β*-mercaptoethanol, 1 mM EGTA, 1 mM NAF, 0.5 mM Na_3_VO_4_, 10 mM *β*-glycerophosphatase and 1 x complete protease inhibitor tablet diluted 1:100), beads were ressuspended in 40 *μ*l master mix (25 mM hepes pH 7.5, 0.1 *β*-mercaptoethanol, 1 mM EGTA, 20 mM magnesium acetate, 20 mM MnCl_2_, 1 mM NAF, 0.5 mM Na_3_VO_4_, 10 mM *β*-glycerophosphatase and 1 x complete protease inhibitor tablet diluted 1:100) plus 10 *μ*l GST-hMLKL-Flag elution and 200 *μ*M adenosine triphosphate. Samples were incubated for 1 h at 30°C with shaking (1200 r.p.m.). Reactions were stopped with 25 *μ*l of standard 5X SDS-sample buffer and heating at 95 °C for 5 min. In all, 20 *μ*l of the supernatant was loaded on an 8% SDS-PAGE gel, and immunoblots probed with human p-MLKL (1:1000, ab187091, Abcam plc) and Flag (M2, Sigma-Aldrich Co.) antibodies.

### Terminal deoxynucleotidyl transferase dUTP nick end labeling (TUNEL) assay and caspase activity assay

TUNEL assay was performed in tissue cryosections (4 *μ*m) using the ApopTag Fluoescein *In Situ* Apoptosis Kit, according to the manufacturer's instructions (Merck Millipore, Darmstadt, Germany). Nuclei were counterstained with Hoechst 33258 (Sigma-Aldrich Co.) at 50 *μ*g/ml in PBS for 6 min at room temperature. Eight images per sample were obtained and fluorescent red nuclei were considered TUNEL-positive cells. Data are expressed as the number of TUNEL-positive cells per mm^2^. Caspase-3/-7 activity was measured using the Caspase-Glo 3/7 Assay (Promega Corp., Madison, WI, USA). Briefly, 15 *μ*g of total protein extracts was incubated with the reagent 30 min at room temperature protected from the light. Luminescence was measured in the GloMax-Multi+ Detection System (Promega Corp.).

### Quantitative RT-PCR

RNA was extracted from animal liver samples using the TRIzol reagent according to the manufacturer's instructions (Thermo Fisher Scientific, Inc.). Total RNA was converted into cDNA using NZY Reverse Transcriptase (NZYTech, Lisbon, Portugal) according to the manufacturer's instructions. Real-time RT-PCR was performed in an Applied Biosystems 7300 System (Thermo Fisher Scientific, Inc.). The following primer sequences were used: for the MIP-2 gene, 5′-GCTACGAACTGCCTGACGG-3′ (forward) and 5′-GCTGTTATAGGTGGTTTCGTGGA-3′ (reverse); for the TLR4 gene 5′-TCCCTGCATAGAGGTAGTTCCTA-3′ (forward) and 5′-CTTCAAGGGGTTGAAGCTCAG-3′ (reverse); for the IL-1*β* gene, 5′-TGCCACCTTTTGACAGTGATG-3′ (forward) and 5′-TGATGTGCTGCTGCGAGATT-3′ (reverse); for the *α-*SMA gene 5′-GCTACGAACTGCCTGACGG-3′ (forward) and 5′-GCTGTTATAGGTGGTTTCGTGGA-3′ (reverse); for the collagen-1*α*1 gene 5′-CTGACTGGAAGAGCGGAGAG-3′ (forward) and 5′-GACGGCTGAGTAGGGAACAC-3′ (reverse); for TGF-*β* gene 5′-CTGCTGACCCCCACTGATAC-3′ (forward) and 5′-GTGAGCGCTGAATCGAAAGC-3′ (reverse); for the HO-1 gene 5′-CGGGCAGCAACAAAGTG-3′ (forward) and 5′-AGTGTAAGGACCCATCGGAGAA-3′ (reverse); for the iNOS gene 5′-ACATCGACCCGTCCACAGTAT-3′ (forward) and 5′-CAGAGGGGTAGGCTTGTCTC-3′ (reverse); for the hepcidin gene 5′-AGGTGACACTATAGAATAGAGAGACACCAACTTCCCCA-3′ (forward) and 5′-GTACGACTCACTATAGGGATCAGGATGTGGCTCTAGGCT-3′ (reverse); for the FtH gene 5′-CCATCAACCGCCAGATCAAC-3′ (forward) and 5′-GCCACATCATCTCGGTCAAA-3′ (reverse); for the FtL gene 5′-AGGTGACACTATAGAATAAAGATGGGCAACCATCTGAC-3′ (forward) and 5′-GTACGACTCACTATAGGGAGCCTCCTAGTCGTGCTTGAG-3′ (reverse); and for the hypoxanthine phosphoribosyltransferase (HPRT) gene 5′-GGTGAAAAGGACCTCTCGAAGTG-3′ (forward) and 5′-ATAGTCAAGGGCATATCCAACAACA-3′ (reverse). Two independent reactions for each primer set were performed in a total volume of 12.5 *μ*l containing 2 × Power SYBR green PCR master mix (Thermo Fisher Scientific, Inc.) and 0.6 *μ*M of each primer. The relative amounts of each gene were calculated based on the standard curve normalized to the level of HPRT and expressed as fold change from sham WT control. To quantitate the relative amounts of RIP3, MLKL and RIP1, total RNA was converted into cDNA using High-Capacity RNA-to-cDNA Kit (Thermo Fisher Scientific, Inc.) according to the manufacturer's instructions. Real-time RT-PCR reaction was performed using the TaqMan Gene Expression Assay (Thermo Fisher Scientific, Inc.) following the manufacturer's instructions. *β*-2-microglobulin (B2M) was used as the normalization control. Relative amounts of each gene were determined by the threshold cycle (2^−ΔΔCt^) method, where ΔΔCt=(Ct_RIP3_– Ct_B2M_) sample – (Ct_RIP3_ – Ct_B2M_) calibrator.

### Analysis of lipid peroxidation and total ROS

The amount of aldehyde end products of lipid peroxidation, namely malondialdehyde was analyzed in whole-liver lysates using a commercial kit (Lipid Peroxidation (MDA) Assay Kit; Sigma-Aldrich Co.). Fluorescence was measured using the GloMax-Multi+ Detection System and values were adjusted to total protein concentration. ROS levels were analyzed through the use of 2′,7′-dichlorodihydrofluorescein diacetate (H_2_DCFDA) (Sigma-Aldrich Co.), a cell-permeant nonfluorescent molecule that is oxidized by ROS to form dichlorofluorescein, a fluorescent compound. Twenty-five mg of liver tissue was homogenized using a glass dounce on 500 *μ*l of ice-cold PBS. To remove insoluble particles, the lysate was centrifuged at 10 200 *g* for 5 min at 4 °C and the supernatant recovered. Fifty *μ*l of the lysate was incubated with 10 *μ*M of H_2_DCFDA at room temperature for 30 min protected from light. The emission of green fluorescence was measured using the GloMax-Multi+ Detection System and values were corrected with total protein content.

### Cell culture and treatments

Primary mouse hepatocytes were isolated from female WT and RIP3^−/−^ mice as previously described.^47, 48^ After isolation, hepatocytes were ressuspended in Complete William's E medium (Sigma-Aldrich Co.) and plated on Primaria tissue culture dishes (BD Biosciences, San Jose, CA, USA) at 5 × 10^4^ cells/cm^2^. Cells were maintained at 37°C in a humidified atmosphere of 5% CO_2_ for 4 h, to allow attachment. Primary mouse hepatocytes were then pretreated with 10 *μ*M of CoPP IX (Santa Cruz Biotechnology Inc., Dallas, TX, USA), an HO-1 inducer, 15 *μ*M ZnPP-9 (Santa Cruz Biotechnology Inc.), a HO-1 inhibitor, 100 *μ*M Nec-1 (Sigma-Aldrich Co.), a necroptosis inhibitor, or dimethyl sulfoxide (Sigma-Aldrich Co.), vehicle control (final concentration of 0.1%). After 1 h, cells were exposed to 50 *μ*M sodium glycochenodeoxycholate (Sigma-Aldrich Co.) or vehicle control. After 24 and 48 h, cells were harvested for total iron assay and cell death assay, respectively.

### Iron measurement

Total iron was measured in murine livers as previously described.^49^ Briefly, liver pieces were dried for 24 h at 95 °C and then dissolved in 3 M HCl/10% trichloracetic acid overnight at 65 °C (0.1 g dried tissue/1 ml). Twenty *μ*l of each homogenized was diluted in 580 *μ*l of water and 10 *μ*l of *β*-mercaptoethanol, 500 *μ*l of 3 M sodium acetate, pH 4.5, and 80 *μ*l bathophenanthroline-disulfonic acid disodium salt hydrate at 1.5 mg/ml were added to each sample. Next, samples were incubated for 1 h at 37 °C and absorbance was measured at 535 nm. The quantity of iron was normalized by the dry tissue mass. This assay measures primarily labile iron, although we cannot exclude residual heme detection. Total iron (ferric and ferrous iron) was measured in whole-cell lysates using a commercial kit (Iron Assay Kit; Sigma-Aldrich Co.), according the manufacturer's instructions. Absorbance was measured using a Bio-Rad model 680 microplate reader (Bio-Rad Laboratories) and values were normalized with total protein concentration.

### Cell death assays

The percentage of general cell death was evaluated using the lactate dehydrogenase (LDH) Cytotoxicity Detection Kit^PLUS^ (Roche Diagnostics GmbH), following the manufacturer's instructions. Experimental LDH values were normalized with maximum releasable LDH activity in the cells, after cell disruption with the provided lysis solution. Hoechst 33258 (Sigma-Aldrich Co.) labeling of attached cells was used to detect apoptotic nuclei by morphological analysis, as previously described.^50^

### Immunofluorescence, immunohistochemistry and image analysis

Paraffin-embedded human liver sections were deparaffined, rehydrated and boiled three times in 10 mM citrate buffer, pH 6. For immunofluorescence, sections were then incubated with 0.1 M glycine in PBS for 10 min and then for 1 h in blocking buffer, containing 0.3% Triton X-100 (Sigma-Aldrich Co.), 1% FBS (Thermo Fisher Scientific, Inc.) and 10% normal donkey serum (Jackson ImmunoResearch Laboratories, Inc., West Grove, PA, USA). Sections were then incubated with a primary antibody reactive to RIP3 (1 : 50; sc-135170, Santa Cruz Biotechnology Inc.) or to p-MLKL (1 : 250, ab187091, Abcam plc) or MLKL (1 : 100, ab183770, Abcam plc), overnight at 4 °C. After rinsing, the primary antibody was developed by incubating with a 1 : 200 secondary Alexa Fluor 594-conjugated anti-rabbit antibody (Thermo Fisher Scientific, Inc.) for 2 h at room temperature. A double staining was performed using 0.05% Thioflavin T (Sigma-Aldrich, Co) solution in PBS for 8 min at room temperature. Nuclei were stained with Hoechst 33258 at 50 *μ*g/ml in PBS for 6 min at room temperature. Samples were mounted using Fluoromount-G (Beckman Coulter, Inc., Brea, CA, USA). Detection of RIP3, p-MLKL and thioflavin T was visualized using an Axio Scope.A1 microscope (Carl Zeiss Microscopy GmbH, Jena, Germany). Images were acquired using an AxioCam HRm camera with the AxioVision software (release 4.8; Carl Zeiss Microscopy GmbH). Semiquantitative analysis of mean fluorescence intensities of RIP3, p-MLKL and thioflavin T and colocalization analysis were performed using the NIH Image J software (Bethesda, MD, USA). Eight images per sample were obtained. Images were converted into an 8-bit format, and the background was subtracted. An intensity threshold was set and kept constant for all images analyzed. For immunohistochemistry, after deparaffinization/rehydration and antigen retrieval, endogenous peroxidase was inactivated by incubating sections in 3% hydrogen peroxide for 10 min at room temperature. Subsequently, sections were incubated for 1 h in blocking buffer, containing 10% normal donkey serum (Jackson ImmunoResearch Laboratories, Inc.) at room temperature. Next, sections were incubated overnight at 4 °C with primary rabbit antibody reactive to p-MLKL (1 : 250, Abcam plc). Detection of the primary antibody was performed using the HiDef Detection HRP Polymer System (Cell Marque, Rocklin, CA, USA), according to the manufacturer's instructions. Sections were developed using SIGMAFAST 3,3′-diaminobenzidine tablets (Sigma-Aldrich Co.) and were counterstained with Mayer's hematoxylin. Finally, slides were rinsed, dehydrated and a glass coverslip was mounted using Entellan mounting media (Merck Millipore). The specimens were examined using a bright-field microscope using an Axio Scope microscope (Zeiss Axioskop; Carl Zeiss GmbH, Jena, Germany). Images were acquired using a DFC490 camera (Leica Microsystems AG, Heersbrugg, Switzerland) with the Zen Lite 2012 (blue edition) for image acquisition (version 1.1.2.0, Carl Zeiss Microscopy GmbH). Negative controls with the omission of the primary antibody were performed in order to validate the results.

### Densitometry and statistical analysis

The relative intensities of protein bands were analyzed using the Image Lab densitometric analysis program (version 5.1; Bio-Rad Laboratories). Statistical analysis included Student's *t*-test when appropriate or one-way analysis of variance with Bonferroni *post hoc* testing when more than two groups were compared. Values of *P*<0.05 were considered statistically significant. All statistical analysis was performed with GraphPad Prism 5 software (GraphPad Software, Inc., San Diego, CA, USA).

## Figures and Tables

**Table 1:** Clinical chemistry parameters

**Metabolite**	**Sham**		**BDL day 3**		**BDL day 14**	
	**WT**	**RIP3**^−/−^	**WT**	**RIP3**^−/−^	**WT**	**RIP3**^−/−^
AST (U/l)	128±34	128±26	760±60*	424±101^*†^	397±104*	555±136*
ALT (U/l)	26±6	23±2	514±40*	295±63^§†^	352±80*	562±88*
AP (U/l)	47±12	23±15	388±87*	253±41*	750±175*	1572±255^§†^
Conj. bilirubin (mg/dl)	0.05±0.01	0.05±0.01	5.0±1.5^§^	4.8±1.2^§^	5.4±2.0^§^	11.4±2.5^§†^
Total bilirubin (mg/dl)	0.12±0.01	0.21±0.06	5.5±1.6^§^	5.9±1.6^§^	8.2±2.3^§^	13.9±1.9*

Abbreviations: ALT, alanine aminotransferase; AP, alkaline phosphatase; AST, aspartate aminotransferase; Conj. bilirubin, conjugated bilirubin. ^§^*P*<0.05 and *P<0.01 from sham-operated mice; ^†^*P*<0.05 from BDL WT mice at respective time-point

## Abbreviations

ALT: alanine aminotransferase
AP: alkaline phosphatase
AST: aspartate aminotransferase
BDL: common bile duct ligation
CoPP: cobalt protoporphyrin
DILI: drug-induced liver injury
FtH: ferritin heavy chain
FtL: ferritin light chain
GCDCA: glycochenodeoxycholic acid
H&E: hematoxylin and eosin
HO-1: heme oxygenase-1
iNOS: inducible nitric oxide synthase
LDH: lactate dehydrogenase
MLKL: mixed lineage kinase domain
NASH: non-alcoholic stetohepatitis
Nec-1: necrostatin-1
PBC: primary biliary cholangitis
p-MLKL: phosphorylated mixed lineage kinase domain
RIP: receptor-interacting protein
RIP3^−/−^: RIP3-deficient mice
ROS: reactive oxygen species
TGF-*β*: transforming growth factor-*β*
TLR4: Toll-like receptor 4
WT: wild type
ZnPP: zinc protoporphyrin-9
