# Supplementary figures and images for: Activation of necroptosis in human and experimental cholestasis
Source: Cell Death Dis. 2016 Sep 29;7(9):e2390–. doi: 10.1038/cddis.2016.280 (PMC5059878; doi:10.1038/cddis.2016.280)

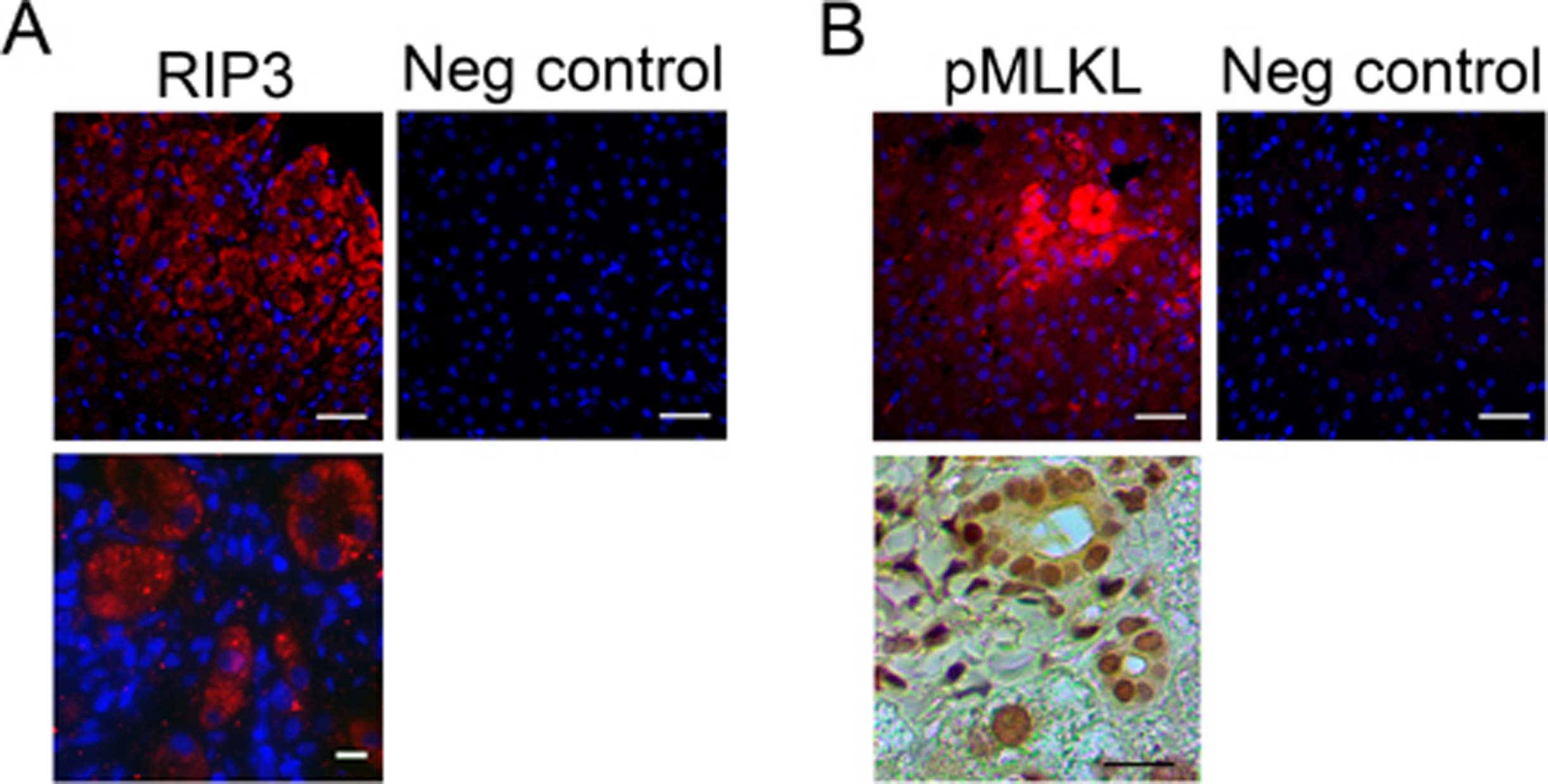

Supplement: Supplementary Figure 1 [file cddis2016280x1.tif]

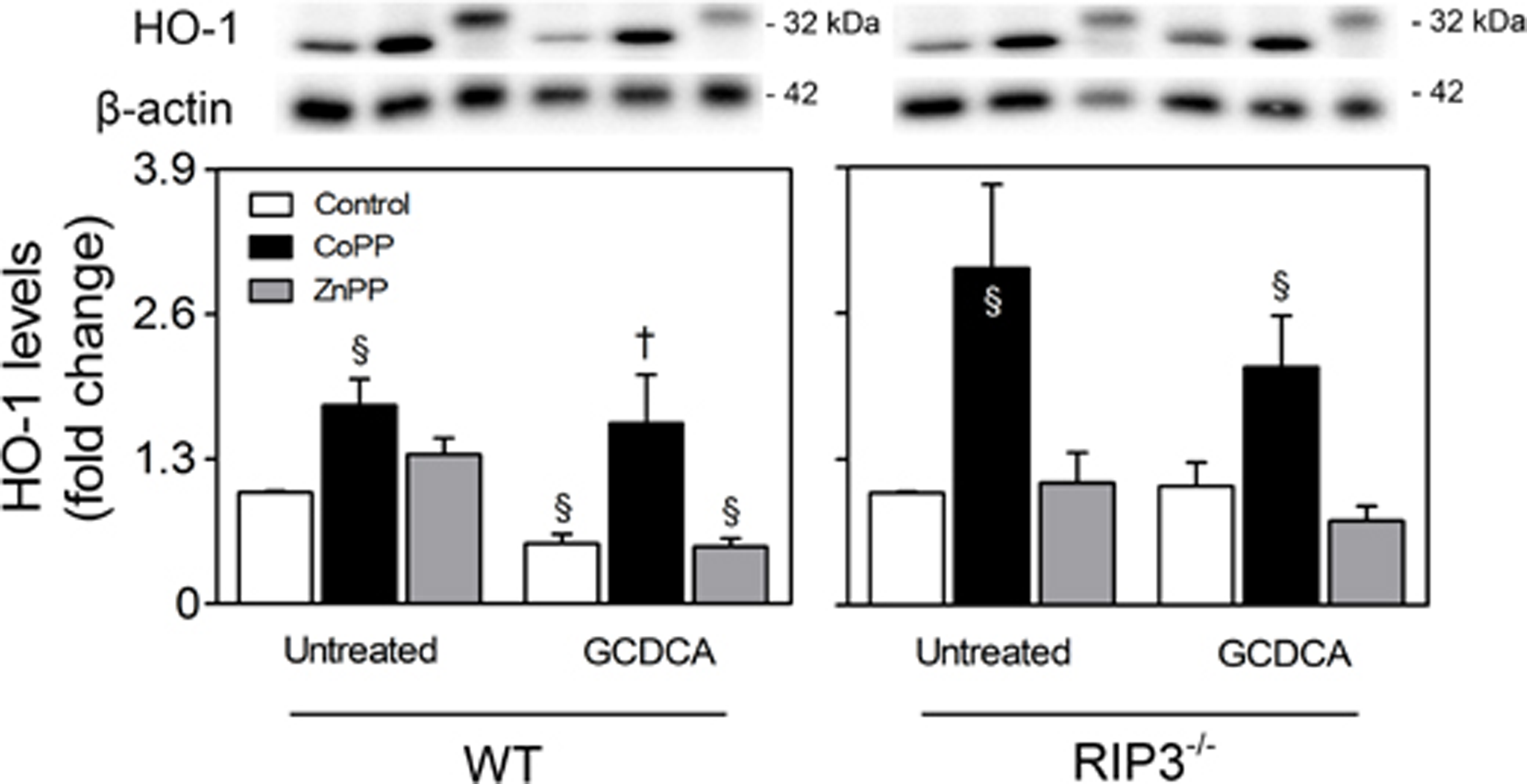

Supplement: Supplementary Figure 2 [file cddis2016280x2.tif]
